# Target product profiles: tests for tuberculosis treatment monitoring and optimization

**DOI:** 10.2471/BLT.23.290901

**Published:** 2023-10-04

**Authors:** Ankur Gupta-Wright, Saskia den Boon, Emily L MacLean, Daniela Cirillo, Frank Cobelens, Stephen H Gillespie, Mikashmi Kohli, Morten Ruhwald, Rada Savic, Grania Brigden, Mustapha Gidado, Delia Goletti, Debra Hanna, Rumina Hasan, Cathy Hewison, Kobto G Koura, Christian Lienhardt, Patrick Lungu, Timothy D McHugh, Lindsay McKenna, Cherise Scott, Thomas Scriba, Christine Sekaggya-Wiltshire, Tereza Kasaeva, Matteo Zignol, Claudia M Denkinger, Dennis Falzon

**Affiliations:** a Heidelberg University Hospital, German Center of Infection Research, Heidelberg, Germany.; b Global Tuberculosis Programme, World Health Organization, 20 Avenue Appia, 1211 Geneva, Switzerland.; c Central Clinical School, University of Sydney, Sydney, Australia.; d San Raffaele Institute, Milan, Italy.; e Amsterdam Institute for Global Health and Development, Amsterdam, Kingdom of the Netherlands.; f Division of Infection and Global Health, University of St Andrews, St Andrews, Scotland.; g FIND, Geneva, Switzerland.; h University of California San Francisco, San Francisco, California, United States of America (USA).; i The Global Fund to Fight AIDS, Tuberculosis and Malaria, Geneva, Switzerland.; j KNCV TB Plus, the Hague, Kingdom of the Netherlands.; k Translational Research Unit, National Institute for Infectious Diseases L Spallanzani-IRCCS, Rome, Italy.; l Bill & Melinda Gates Foundation, Seattle, USA.; m Aga Khan University, Karachi, Pakistan.; n Médecins Sans Frontières, Paris, France.; o The International Union Against Tuberculosis and Lung Disease, Paris, France.; p French National Research Institute for Sustainable Development (IRD), Montpellier, France.; q East, Central and Southern Africa Health Community, Arusha, United Republic of Tanzania.; r Centre for Clinical Microbiology, University College London, London, England.; s Treatment Action Group, New York, USA.; t Unitaid, Geneva, Switzerland.; u University of Cape Town, Cape Town, South Africa.; v Infectious Diseases Institute, Kampala, Uganda.

## Abstract

The World Health Organization has developed target product profiles containing minimum and optimum targets for key characteristics for tests for tuberculosis treatment monitoring and optimization. Tuberculosis treatment optimization refers to initiating or switching to an effective tuberculosis treatment regimen that results in a high likelihood of a good treatment outcome. The target product profiles also cover tests of cure conducted at the end of treatment. The development of the target product profiles was informed by a stakeholder survey, a cost-effectiveness analysis and a patient-care pathway analysis. Additional feedback from stakeholders was obtained by means of a Delphi-like process, a technical consultation and a call for public comment on a draft document. A scientific development group agreed on the final targets in a consensus meeting. For characteristics rated of highest importance, the document lists: (i) high diagnostic accuracy (sensitivity and specificity); (ii) time to result of optimally ≤ 2 hours and no more than 1 day; (iii) required sample type to be minimally invasive, easily obtainable, such as urine, breath, or capillary blood, or a respiratory sample that goes beyond sputum; (iv) ideally the test could be placed at a peripheral-level health facility without a laboratory; and (v) the test should be affordable to low- and middle-income countries, and allow wide and equitable access and scale-up. Use of these target product profiles should facilitate the development of new tuberculosis treatment monitoring and optimization tests that are accurate and accessible for all people being treated for tuberculosis.

## Résumé

L'Organisation mondiale de la santé a élaboré des profils de produits cibles contenant des cibles minimales et optimales pour les caractéristiques principales des essais destinés au suivi et à l'optimisation du traitement de la tuberculose. L'optimisation du traitement de la tuberculose fait référence à l'instauration d'un régime de traitement efficace de la tuberculose ou à l'adoption d'un tel régime, avec une probabilité élevée d'obtenir de bons résultats thérapeutiques. Les profils de produits cibles couvrent également les essais de guérison effectués à l'issue du traitement. Les profils de produits cibles ont été élaborés sur la base d'un sondage auprès des parties prenantes, d'une analyse coût-efficacité et d'une analyse du parcours de soins du patient. Des retours supplémentaires des parties prenantes ont été obtenus au moyen d'un processus créé selon la méthode Delphi, d'une consultation technique et d'un appel à commentaires publics sur un projet de document. Un groupe d'élaboration scientifique s'est mis d'accord sur les objectifs finaux lors d'une réunion de concertation. En ce qui concerne les caractéristiques jugées les plus importantes, le document énumère ce qui suit: (i) une grande précision diagnostique (sensibilité et spécificité); (ii) un délai idéal d'obtention des résultats ≤ 2 heures et au maximum de 1 jour; (iii) le type d'échantillon requis doit être peu invasif et facile à obtenir, comme l'urine, l'haleine ou le sang capillaire, ou bien un échantillon respiratoire au-delà des expectorations; (iv) idéalement, l'essai pourrait avoir lieu dans un établissement de santé périphérique sans laboratoire ; et (v) l'essai devrait être abordable pour les pays à revenu faible et intermédiaire et permettre un accès large et équitable ainsi qu'une mise à l'échelle. L'utilisation de ces profils de produits cibles devrait faciliter la mise au point de nouveaux essais de surveillance et d'optimisation du traitement de la tuberculose qui soient précis et accessibles à toutes les personnes suivant un traitement pour la tuberculose.

## Resumen

La Organización Mundial de la Salud ha elaborado perfiles de productos objetivo que contienen objetivos mínimos y óptimos para las características principales de las pruebas de seguimiento y optimización del tratamiento de la tuberculosis. La optimización del tratamiento de la tuberculosis consiste en iniciar o cambiar a un régimen eficaz de tratamiento de la tuberculosis que ofrezca una alta probabilidad de un buen resultado terapéutico. Los perfiles de productos objetivo también abarcan las pruebas de curación realizadas al final del tratamiento. La elaboración de los perfiles de los productos objetivo se basó en una encuesta a las partes interesadas, un análisis de rentabilidad y un análisis de la vía de atención al paciente. Se obtuvo información adicional de las partes interesadas mediante un proceso tipo Delphi, una consulta técnica y una convocatoria de comentarios públicos sobre un borrador del documento. Un grupo de desarrollo científico acordó los objetivos finales en una reunión de consenso. Para las características clasificadas de mayor importancia, el documento enumera: (i) alta precisión diagnóstica (sensibilidad y especificidad); (ii) tiempo hasta el resultado de óptimamente ≤ 2 horas y no más de 1 día; (iii) el tipo de muestra requerida debe ser mínimamente invasiva, fácil de obtener, como orina, aliento o sangre capilar, o una muestra respiratoria que vaya más allá del esputo; (iv) idealmente la prueba podría realizarse en un centro sanitario periférico sin laboratorio; y (v) la prueba debe ser asequible para los países de ingresos bajos y medios y permitir un acceso amplio y equitativo y su expansión. El uso de estos perfiles de producto objetivo debería facilitar el desarrollo de pruebas nuevas de seguimiento y optimización del tratamiento de la tuberculosis que sean precisas y accesibles para todas las personas que reciben tratamiento antituberculoso.

## ملخص

قامت منظمة الصحة العالمية بتصميم ملفات تعريف المنتجات المستهدفة التي تحتوي على كل من الحد الأدنى والأهداف المثلى للخصائص الرئيسية لاختبارات مراقبة علاج السل وتحسينه. يشير تحسين علاج السل إلى بدء، أو التحول إلى، نظام فعال لعلاج السل يؤدي إلى احتمال كبير للحصول على نتائج علاجية جيدة. تغطي ملفات تعريف المنتجات المستهدفة أيضًا اختبارات العلاج التي يتم إجراؤها في نهاية العلاج. اعتمد تطوير تصميم ملفات تعريف المنتجات المستهدفة على مسح لأصحاب المصلحة، وتحليل لفعالية التكلفة، وتحليل لمسار رعاية المرضى. تم الحصول على تعليقات إضافية من أصحاب المصلحة من خلال عملية تشبه تقنية Delphi، والاستشارات الفنية، والدعوة للتعليق العام على وثيقة مبدئية. وافقت مجموعة للتطوير العلمي على الأهداف النهائية في اجتماع بتوافق الآراء. بالنسبة للخصائص المصنفة ذات الأهمية القصوى، تسرد الوثيقة ما يلي: (1) دقة تشخيصية عالية (الحساسية والنوعية)؛ و(2) الوقت المثالي للحصول على النتيجة هو أقل من أو يعادل ساعتين، ولا يزيد عن يوم واحد؛ و(3) يجب أن تكون العينة المطلوبة من النوع محدود الجراحة، ويمكن الحصول عليه بسهولة، مثل البول أو التنفس أو دم الشعيرات الدموية، أو عينة من الجهاز التنفسي تتجاوز البلغم؛ و(4) بشكل مثالي، يمكن إجراء الاختبار في منشأة صحية على مستوى ملائم دون الحاجة لمختبر؛ و(5) ينبغي أن يكون الاختبار ميسور التكلفة بالنسبة للدول ذات الدخل المنخفض والمتوسط، ومتاح إجراؤه على نطاق واسع وعادل وواسع النطاق. يجب أن يؤدي استخدام ملفات تعريف المنتجات المستهدفة هذه إلى تسهيل تطوير اختبارات جديدة لمراقبة علاج السل وتحسينه، بحيث تكون دقيقة ويمكن لجميع الأشخاص الذين يعالجون من مرض السل الحصول عليها.

## 摘要

世界卫生组织制定了目标产品简介，其中包含结核病治疗监测和优化测试的关键特征的最低和最佳目标。结核病治疗优化是指开始或改用有效的结核病治疗方案，因此获得良好治疗结果的可能性很高。目标产品简介还包括在治疗结束时进行的临床疗效测试。目标产品简介的开发以利益攸关者调查、成本效益分析和患者护理途径分析为信息依据。通过类似德尔菲法 (Delphi-like process)、技术咨询和对文件草案征求公众意见的方式，获得了利益攸关者的更多反馈。一个科学发展小组在一次共识会议上商定了最终目标。该文档列出的被评为最重要的特征如下：(i) 诊断准确性高（敏感性和特异性）；(ii) 获得最佳结果的时间不超过 2 个小时，最多不超过 1 天；(iii) 所需样本类型为微创且易于获得的样本，如尿液、呼吸道或毛细血管血液，或除痰液以外的呼吸道样本；(iv) 理想情况下，测试可以在没有实验室的外围卫生机构进行；以及(v) 测试应该是低收入和中等收入国家可以负担得起的，并能够大范围、公平合理和大规模的进行。使用这些目标产品简介应有助于开发新的结核病治疗监测和优化测试，这些测试对所有接受结核病治疗的人来说都是准确且易于使用的。

## Резюме

Всемирной организацией здравоохранения разработаны целевые профили продукции, содержащие минимальные и оптимальные значения ключевых характеристик для анализов, предназначенных для мониторинга и оптимизации лечения туберкулеза. Под оптимизацией лечения туберкулеза понимается начало или переход на эффективную схему лечения туберкулеза, обеспечивающую высокую вероятность благоприятного исхода лечения. Целевые профили продукции также охватывают оценку излечения, проводимую по окончании лечения. При разработке целевых профилей продукции были использованы результаты опроса заинтересованных сторон, анализ экономической эффективности и анализ планов ведения пациентов. Дополнительные отзывы заинтересованных сторон были получены путем использования процесса, аналогичного дельфийскому методу (методу экспертных оценок), технических консультаций и обращений к общественности с просьбой предоставить комментарии к проекту документа. Окончательные целевые показатели согласованы группой научной разработки в ходе совещания по выработке консенсуса. В качестве характеристик, оцениваемых как наиболее важные, в документе приводятся: (i) высокая точность диагностики (чувствительность и специфичность); (ii) время получения результата – оптимально ≤ 2 часов и не более 1 дня; (iii) возможность получения нужного типа образца путем минимального инвазивного вмешательства, например моча, выдыхаемый воздух или капиллярная кровь, либо дыхательные пробы, не ограничивающиеся мокротой; (iv) в идеале анализ должен допускать выполнение в медицинских учреждениях периферийного уровня без лаборатории; (v) анализ должен быть доступен по цене для стран с низким и средним уровнем дохода и обеспечивать широкий и равный доступ с расширением масштабов. Использование таких целевых профилей продукции должно способствовать разработке новых анализов, предназначенных для мониторинга и оптимизации лечения туберкулеза, которые будут точными и доступными для всех пациентов, получающих лечение от туберкулеза.

## Introduction

Tuberculosis continues to be a major cause of morbidity and mortality globally, despite being curable and preventable. In 2021, an estimated 10.6 million people had tuberculosis disease and 1.6 million people died from it.^1^ Tuberculosis treatment regimens are long and arduous and can be associated with adverse effects and significant financial costs, which negatively affect adherence and treatment outcomes.^2^ Worldwide, only 86% of people who started on tuberculosis treatment in 2020 successfully completed it.^1^

Monitoring tuberculosis treatment to identify those at risk of poor outcomes could improve overall treatment success. The World Health Organization (WHO) currently recommends sputum smear microscopy and mycobacterial culture to monitor the response to treatment in pulmonary tuberculosis two months after treatment initiation, and during the last month of treatment as a test of cure.^2^^–^^6^ However, these methods have limitations. First, they are dependent on sputum samples which are often not available (especially in people with extrapulmonary tuberculosis, children and people living with human immunodeficiency virus, HIV). Second, there is a poor diagnostic accuracy of smear microscopy for predicting treatment outcome.^7^ Finally, there are high infrastructure requirements and costs, slow turn-around time of culture and poor sensitivity to predict relapse.^8^^,^^9^ Even combined with other indicators, such as weight, clinical symptoms and chest radiographs, performance is poor and there remains a need for accurate tests to identify people who have been cured of tuberculosis.^10^^,^^11^ Therefore, WHO formulated target product profiles to provide product characteristics that should be considered during the development of new tests for tuberculosis treatment monitoring and optimization.^12^

Tuberculosis treatment optimization refers to initiating or switching to an effective tuberculosis treatment regimen that results in a high likelihood of a good treatment outcome. Optimization includes stratifying treatments at initiation, and adapting treatment duration or applying adjuvant interventions based on disease severity. A stratified approach to tuberculosis treatment, where treatment is tailored to disease severity, might be possible in the future^13^^,^^14^ and is already recommended for children and young adolescents: WHO guidelines include a 4-month regimen for non-severe tuberculosis.^15^^,^^16^ Optimization also includes adjusting treatment in patients with poor response to the treatment regimen being used, for example due to more severe disease, drug resistance, poor adherence or malabsorption. 

A treatment monitoring test should indicate poor response to treatment and identify those who would benefit from further assessment, such as drug susceptibility testing or adherence monitoring and support, to inform potential changes to tuberculosis treatment or management. The target product profiles also cover tests of cure, conducted at the end of treatment to indicate that an individual does not require further treatment. The target product profiles apply to the monitoring and optimization of tuberculosis treatment regimens for drug-susceptible tuberculosis and drug-resistant tuberculosis, but exclude drug-resistance testing, for which separate target product profiles are available.^17^

The target audience for these target product profiles includes commercial test developers and manufacturers, academia and research institutions, donors, regulatory agencies, nongovernmental organizations, private sector implementers, national tuberculosis programmes, civil society organizations and people with tuberculosis.

## Methods

In 2022, WHO established a scientific target product profiles development group, consisting of scientists and experts, public health specialists, donors and civil society representatives. Members of the development group were engaged throughout the development process, and proposed the final target product profiles during an in-person meeting. All members made a declaration on potential conflicts of interest, which were reviewed and managed by WHO; a statement on the declarations is available in the WHO target product profiles document.^12^ A subset of the development group members created a task force and was consulted more often to guide the development of the target product profile. The task force prepared a first working draft of the target product profile document based on two recent literature reviews, a draft target product profile (unpublished data, FIND, 2015) and multiple meetings of the task force.^18^^,^^19^

WHO administered an online survey to obtain opinions from a wide range of stakeholders, including test developers, researchers, staff of national tuberculosis programmes, laboratory scientists, implementers and clinicians, and representatives from industry and civil society organizations. The survey included questions about the perceived need for the target product profiles; use cases, definitions and aims; target patient populations; and key characteristics. If survey participants disagreed with the proposed definitions or characteristics, they were asked to comment and to provide a suggestion for improvement. The draft target product profiles were revised based on the responses to the survey.

In parallel, two studies were conducted to inform the development of the target product profiles: (i) mapping of patient-care pathways, describing the main clinical decisions associated with treating individual people with tuberculosis, which was informed by a literature and guideline review, and a survey of national tuberculosis programmes and others who support tuberculosis treatment programmes in high-burden countries; and (ii) cost-effectiveness modelling to explore the potential health impacts and costs of hypothetical novel treatment monitoring tests. These two studies will be published separately.

The draft document was reviewed during a technical consultation with development group members and additional stakeholders, including product developers. The Delphi method was used to help reach agreement on proposed targets: a survey was administered before the meeting and the results informed and guided the discussions during the technical consultation in September 2022. Changes and suggestions from the Delphi process and technical consultation were incorporated into a further refined draft document.

The draft document was subsequently posted on the WHO website for a month to solicit public feedback, specifically inviting relevant stakeholders and interested parties to provide feedback by March 2023.^20^ The results of the public consultation were presented at a final development group meeting, where consensus was reached on the final target product profiles.

For each characteristic, minimal and optimal targets are defined. Minimal refers to the lowest acceptable output for a characteristic, and optimal reflects a realistically achievable ideal target for that characteristic. The expectation is that new tests would meet most of the minimal requirements and as many of the optimal requirements as possible.

## Target product profiles^12^

Based on key decisions facing clinicians in the management of people with tuberculosis, the development group identified three main use cases for tuberculosis treatment monitoring and optimization tests. These are: (i) treatment initiation: tests used at treatment initiation to identify people with tuberculosis who require a more intensive treatment regimen; (ii) treatment monitoring: tests used to identify people at risk of a poor treatment outcome during tuberculosis treatment; and (iii) treatment outcome: tests used to identify people with a poor treatment outcome at the end of tuberculosis treatment (Table 1). Targets regarding accuracy, test timing and frequency specific for each use case were developed. In addition, the profiles contain operational characteristics that are common to all tests. Further information and tabular format of the text presented below is available in the published target product profile document.^12^

**Table 1:** Use cases for tuberculosis treatment monitoring and optimization tests

Use case	Timing	Explanatory notes	Consequences
Identify people who require a more intensive tuberculosis treatment regimen	Treatment initiation	Prioritizes avoiding undertreatment of people who would have a poor treatment outcome^a^ on a less intensive regimen (e.g. those with more severe disease)	If the test predicts a poor treatment outcome, the person will be started on a more intensive tuberculosis regimen.^b^ If the test predicts a good treatment outcome,^c^ the person can be initiated on a less intensive tuberculosis regimen^d^ (albeit with ongoing monitoring)
Identify people at risk of a poor outcome with current tuberculosis treatment	During treatment	Aims to identify people who are not adequately responding to tuberculosis treatment. These are sometimes known as tests for tuberculosis treatment monitoring or treatment response, and they have aims similar to using sputum-based microscopy or culture during treatment	If the test shows high risk of poor treatment outcome, the person may need a different, more intensive optimized treatment regimen, may need to undergo further testing or may need adherence support interventions. If a test shows a good treatment response, the person can continue with the current treatment
Identify people with a poor treatment outcome at the end of tuberculosis treatment	Presumed end of treatment	Aims to identify those who have a poor treatment outcome or are at risk of early relapse (i.e. as is currently done using microscopy or culture). These tests need to be accurate enough to not miss people who are not cured or who will relapse early, and also to minimize the number of people incorrectly identified as not cured. These are sometimes called tests of cure	If the test shows a poor treatment outcome or high risk of early relapse, the person may require further investigations, continuing or optimizing of the treatment regimen, or a combination of these. If the test shows a good treatment outcome has been achieved, the current regimen can be considered completed

WHO: World Health Organization.

^a^ Poor outcome (or poor treatment outcome) is a lack of bacteriological or clinical improvement, or both, by the end of tuberculosis treatment; early relapse; the need to prematurely terminate or switch tuberculosis treatment; or death related to tuberculosis.

^b^A more intensive tuberculosis treatment regimen may be longer, contain more medicines or include adjuvant therapies.

^c^ Good outcome (or good treatment outcome) is bacteriological or clinical improvement, or both, at the end of treatment for tuberculosis without evidence of relapse within 6 months. This definition is aligned with revised WHO treatment outcome definitions and incorporates the definition of cured (i.e. evidence of bacteriological response) but also includes people without bacteriologically confirmed tuberculosis who have a good clinical response.^6^ This definition also incorporates the operational research definition of sustained treatment success.

^d^ Less intensive tuberculosis treatment regimens may be shorter in duration or contain fewer medicines. It is assumed that these different regimens will be non-inferior to the standard of care in terms of efficacy, but less intensive regimens will have some advantages to people with tuberculosis and tuberculosis programmes (e.g. shorter duration, smaller pill burden, less risk of adverse reactions to medicines).

Note: adapted from *Target product profiles for tests for tuberculosis treatment monitoring and optimization*.^12^

### Treatment initiation 

#### Accuracy

The test should have a high sensitivity as it aims to identify patients at high risk for a poor treatment outcome, ensuring they are not started on less intensive treatment regimens. Ideally, the test should have a sensitivity of 95% or higher. At a minimum, it should have a sensitivity of 90% or higher. Note that some people will have a poor treatment outcome due to unpredictable factors at treatment initiation (e.g. poor adherence). Ideally, the test should also correctly identify those who could achieve a good treatment outcome with less intensive treatment and thus avoid overtreatment. Therefore, the development group decided that the specificity should ideally be 80% or higher, and at a minimum 70% or higher. However, overtreatment is often less problematic, therefore, the profiles prioritize sensitivity over specificity. The accuracy should apply regardless of strain resistance pattern and disease localization.

#### Timing

Tests identifying people who require more intensive tuberculosis regimens should provide results before the patient starts treatment, or at treatment initiation to ensure optimal treatment management from the outset. However, the ideal test would remain accurate if results are provided soon after tuberculosis treatment initiation, especially if people begin treatment before a sample being collected (for example, if treatment is initiated before a patient is referred to tuberculosis services where this test is available). The optimal requirement for providing results was therefore set to up to 7 days after starting treatment.

### Treatment monitoring 

#### Accuracy

The test should identify people with inadequate response to treatment who are likely to have a poor treatment outcome on their current regimen. These people may benefit from further investigation, optimization of tuberculosis treatment or other interventions. The minimal sensitivity target, set at ≥ 75%, is based on the estimated accuracy of the clinical scores for predicting poor outcomes in pulmonary tuberculosis, as well as current treatment monitoring tools.^21^^,^^22^ Ideally, the sensitivity should be 90% or higher.

More intensive tuberculosis treatment regimens are prone to increased adverse events, longer duration and higher costs for patients and health systems. Hence the test should also correctly identify those people who are responding favourably to treatment and are likely to have a good outcome on less intensive treatment. Ideally, the specificity should be 90% or higher, but at least 80% or higher. 

#### Timing and frequency

Results should be available as early as possible during tuberculosis treatment, at a time point when the test has sufficient likelihood of detecting poor treatment outcomes that could be prevented by optimizing tuberculosis treatment. Ideally, results should be provided within 4 weeks after treatment initiation, but the minimum requirement is halfway through the anticipated tuberculosis treatment regimen. From a programmatic perspective, it would be beneficial for the time points to align with routine follow-up appointments. However, the timing of routine follow-up may change with newer and shorter treatment regimens, or improved treatment monitoring tests. Test timing might also depend on the test mechanism. Time at which the test is performed may also impact diagnostic accuracy, as the risk of an undetected poor outcome will be reduced over time. Ideally, the test would be used once at follow-up visit during treatment, without the need for baseline measurement, to reduce health service costs, however tests using two time points for comparison is acceptable. 

### Treatment outcome

#### Accuracy

Ideally, the test should have high sensitivity of 95% or higher to identify people with a poor outcome despite completing their prescribed tuberculosis treatment. At a minimum, it should have a sensitivity of 80% or higher. People identified as having a poor outcome will require further investigations or optimized treatment, or both, and be followed up appropriately.

To reduce overtreatment, the test should ideally have a specificity of 95% or higher, and no less than 90%, to accurately identify those people with a good treatment outcome. Overtreatment carries significant costs and implications for both programmes and patients. The prevalence of poor outcomes will be lower at the end of tuberculosis treatment, which is why the specificity is higher than for the other use cases.

#### Timing and frequency

The test should be done at or just before anticipated completion of tuberculosis treatment to maximize its accuracy for detecting poor treatment outcomes. Ideally, the test would be performed once, either at the final follow-up visit or final medication refill to reduce cost and optimize uptake, but it may need to be done at least twice to compare measurements (for example, earlier during treatment).

### Operational characteristics

#### Assay or instrument design

All tests should ideally be instrument-free, feasible for use at the point-of-care and at the primary care level, where the majority of tuberculosis patients are treated. However, instrument-based tests requiring basic laboratory infrastructure, for example, as found at a district-level hospital, may be acceptable, as long as this approach does not prolong the turnaround time. If the test requires an instrument, this would ideally be compact, be able to be placed in peripheral health facilities and suitable for diagnosing multiple diseases. Furthermore, the instrument should be maintenance free, or maintenance should be done locally or remotely. Yearly servicing of instrument would be acceptable. 

Ideally, tests should be fast, simple to operate and come with an automated reader to minimize impact on workflow. Optimally, up to four tests should be able to run independently from one another. The test should ideally work in up to 45 °C and 90% relative humidity with no power requirements, or at least up to 30 °C and 70% relative humidity. Quality controls that are integrated in the tests would be optimal, however, providing reagents for quality control would be acceptable. The tests should have minimal environmental impact. Non-laboratory-based tests, for example, imaging or clinical scores, may also meet the requirements of the target product profiles.

#### Target placement and user

These tests should ideally be feasible to implement at community health-care facilities, without a laboratory, where people usually undergo follow-up for tuberculosis treatment. At a minimum, the test should be compatible with use in a district hospital-level laboratory. Tuberculosis treatment is mostly monitored by health-care workers with minimal training, including community health workers. Therefore, these tests should ideally be targeted at the same cadre of health-care personnel. The optimal target includes a self-test, whereby people with tuberculosis perform the test and either interpret the results themselves, or contact a health-care worker for interpretation or follow-up action.

The training required to use the test should ideally take one day or less, and at a maximum 3 days. 

#### Target population

Ideally, the test would be applicable to all people starting or already receiving tuberculosis treatment, including children, elderly people, pregnant women, people living with HIV, people with severe malnutrition or comorbidities, and people with drug-resistant tuberculosis or extrapulmonary or disseminated tuberculosis, or both. However, a test only applicable to a subpopulation undergoing tuberculosis treatment may be acceptable, particularly if the test’s nature precludes its use in all people, but provides substantial improvement over existing similar tests and is cost effective.

#### Sample type

Tests should not rely solely on sputum as the sample type, since not everyone with tuberculosis produces it. Moreover, safely obtaining sputum can be challenging, and its availability often diminishes as tuberculosis treatment advances. As a minimal requirement, tests should use a range of respiratory samples. Tests based on minimally invasive, non-sputum respiratory samples, for example, oral swabs, saliva or breath, are likely to have a higher yield than sputum and thus perform better for all people with pulmonary tuberculosis. Ideally, samples should be minimally invasive and easy to access, such as urine or capillary blood. The volume required should be possible to collect with one sample. Additionally, tests could be based on non-samples, for example imaging modalities such as chest radiographs or digital chest radiographs with computer-aided detection or ultrasound, or could be based on scores from multiple clinical observations with or without a sample-based component.

#### Time to result and interpretation

The time between when a sample is received to the release of results should be 2 hours or less under optimal programmatic conditions, however a maximum of 1 day is acceptable. Ideally, results should be available during the same clinical encounter (e.g. on the day of attendance) so that health-care workers can make decisions about management and treatment immediately. In addition, results should be easily interpretable by the target users for the test. Minimally, guidance should be provided to health-care workers on interpretation of quantitative results and their translation into changes in tuberculosis treatment, for example, a cut-off defining a poor response to treatment. Results should be easily available to the health-care worker and digitized for easy sharing with individuals undergoing tuberculosis treatment, for example via short message service, mobile phone or by e-mail.

#### Costs

The cost-effectiveness modelling within these target product profiles suggests that tests meeting the optimal target criteria could prevent 22% (uncertainty interval: 16–29%) of unfavourable treatment outcomes and related disability-adjusted life years that would occur without treatment monitoring. This result underscores the value of investing in such technologies. Based on this modelling, tests meeting the optimal criteria for sensitivity and specificity to detect a poor response to treatment should cost less than United States dollars 25 to achieve better health impacts at lower costs as compared with sputum smear microscopy. However, this cost is indicative only, and it is expected that the price of the test reflects the cost of production, and that public support for development and market shaping would lower costs. Tests must be affordable for tuberculosis programmes, as tests that are not affordable are often not implemented or have poor market penetration, even if cost effective. Costs should also be compatible with ensuring wide and equitable access, and scale-up and affordability is essential for uptake in low-income countries. Furthermore, developers should be aware that costs, resource requirements and issues of equity, acceptability and feasibility are important criteria used by WHO to recommend technologies such as the tests specified in these target product profiles.^23^

### Predictive values

**Fig. 1:**
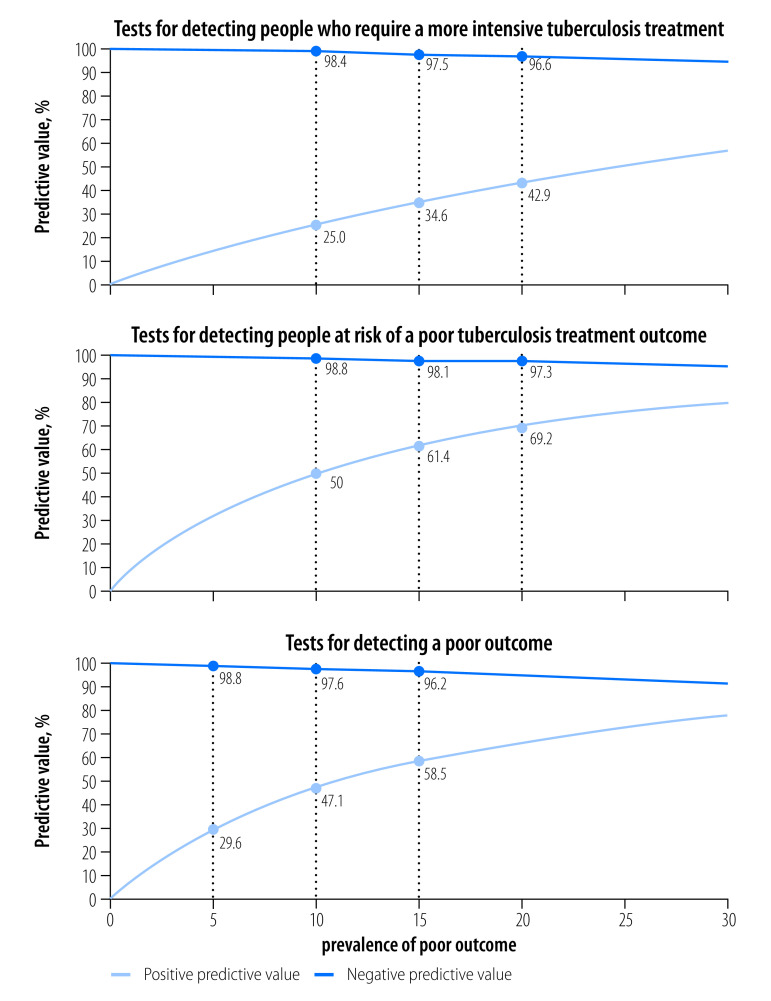
Predictive values of tests for tuberculosis treatment monitoring and optimization

Notes: For detecting people who require a more intensive tuberculosis treatment, we assumed the test had a sensitivity of 90% and specificity of 70% (minimal target at treatment initiation). For detecting those at risk of a poor tuberculosis treatment outcome, we assumed the test had a sensitivity and specificity of 90% (optimal target during treatment). For detecting a poor outcome, we assumed the test had a sensitivity of 80% and specificity of 90% (minimal target at the end of tuberculosis treatment).

The target product profiles^12^ also contain predictive values for each of the three use cases. Although we have outlined diagnostic accuracy targets in terms of sensitivity and specificity, predictive values of tests are more valuable from the patient, clinician and programmatic perspective. These values provide insight into the probability or improbability of a disease state based on a specific test results (Fig. 1).

## Discussion

Here we describe key characteristics for tests for tuberculosis treatment monitoring and optimization. Ideally, one test will be developed that can be used for all three use cases. Developing tests that meet all criteria outlined in the target product profiles might be challenging. Test developers may need to weigh potential trade-offs. For example, developers may prioritize test accuracy even if it necessitates the use of a district-level laboratory. When making such considerations it is important to note that diagnostic accuracy, time to result, sample type, target placement of test and cost are considered priority characteristics due to their potential influence on optimizing treatment and averting unfavourable outcomes.

While many of the characteristics and targets in these target product profiles assume that a test will be biomarker-based, the target product profiles also apply to non-biomarker-based tests such as imaging, scores based on clinical features or assessment of cough sounds. These tests may be more acceptable to people with tuberculosis and tuberculosis programmes, and preferred to laboratory-based tests. The array of emerging tests – capable of monitoring tuberculosis treatment, predicting outcomes, confirming cure and enhancing management – encompasses assays for host characteristics such as cytokines, transcriptomic profiles and other biomarkers. Emerging tests also include assays for pathogen burden and fitness, imaging-based tests, as well as evaluations based on clinical scores, symptoms, signs, cough sounds and lung function tests.^18^^,^^19^ A combination of tests and/or clinical attributes might potentially meet the objectives of the target product profile.

Once products are developed that potentially meet the criteria outlined in these target product profiles, they should undergo evaluation in independent studies. A reference standard is useful when considering the diagnostic accuracy of tests. However, no test is perfect in measuring treatment response or optimization. Therefore, the best reference standard for evaluating these tests may be the treatment outcomes 6 months after the end of treatment. A more detailed discussion of reference standards will be available in a forthcoming article, that provides methodological guidance for evaluating tests for treatment monitoring and optimization (McLean EL et al., University of Sydney, Australia, unpublished data, 2023).

In conclusion, the new WHO target product profiles for tuberculosis treatment monitoring and optimization represent an important statement by global stakeholders about the need for new treatment monitoring and optimization tests. The use of these target product profiles should aid the development of more accurate and highly accessible treatment monitoring and optimization tests for tuberculosis, and is critical to ensure targeted research and development efforts and improve patient outcomes.
